# Risk perception of the pre-distribution of stable iodine to guardians of children living around the Genkai Nuclear Power Plant, Saga Prefecture, Japan

**DOI:** 10.1371/journal.pone.0250570

**Published:** 2021-05-13

**Authors:** Hitomi Matsunaga, Makiko Orita, Yasuyuki Taira, Noboru Takamura

**Affiliations:** Department of Global Health, Medicine and Welfare, Atomic Bomb Disease Institute, Nagasaki University, Nagasaki, Japan; Columbia University, UNITED STATES

## Abstract

Iodine thyroid blocking (ITB) is effective for preventing childhood thyroid cancer when radioactive iodine is released into the environment during a nuclear power plant accident. Japan employs the pre-distribution of stable iodine (PDSI) to residents living near nuclear power plants; however, the number of residents who have actually received stable iodine to date remains limited. The aim of this study was to evaluate the profile of guardians of children living around the Genkai Nuclear Power Plant (GNPP) in Japan. We distributed self-administered questionnaires regarding perception of risks associated with administration of stable iodide to approximated 400 guardians of children aged 0–6 in 10 kindergartens located in four municipalities. We obtained responses from 286 guardians, and after excluding invalid responses, 247 were included in the analysis. Logistic regression analysis revealed that living within 5 km of the GNPP (odds ratio [OR] = 4.48, 95% confidence interval [CI]: 2.43–8.24), awareness of preferential implementation of ITB to children (OR = 3.33, 95%CI: 1.78–6.22), and awareness of the prophylaxis booklet published by the local government (OR = 2.53, 95%CI: 1.37–4.68) were independently associated with PDSI for children. The main reasons for not receiving PDSI were “anxiety about the side effects of stable iodine” (40.2%), “distrust of the effectiveness of SI” (23.5%), “complicated procedures for receiving stable iodine” (15.7%) and “missed the date for receiving stable iodine” (8.8%). In the case of ITB implementation during a nuclear emergency, it is necessary to clarify the risk perceptions of guardians and adapt risk communication accordingly.

## Introduction

Prophylaxis of stable iodine (SI) is a key strategy for reducing the risk of thyroid cancer after consuming foods contaminated by radioactive iodine, such as iodine-131 (^131^I), or after inhaling radioactive iodine during an unexpected nuclear power plant accident [1, 2]. Iodine thyroid blocking (ITB) is effective in minimizing internal exposure to the thyroid, especially in children, adolescents, and pregnant and breastfeeding women living around nuclear facilities [3, 4]; it is less effective in those over 40 years of age [5]. It is well known that after the Chernobyl Nuclear Power Plant accident in 1986, there was a dramatic increase in thyroid cancer among children [6, 7]. Based on the lessons learned from the Chernobyl accident, it is important to implement ITB in children, and to develop a comprehensive plan for evacuation, sheltering, and restrictions on the consumption of contaminated food and water in advance to minimize the exposure doses of residents due to unexpected nuclear disasters [8, 9].

In Japan, after the accident at Tokyo Electric Power Company’s Fukushima Daiichi Nuclear Power Station (FDNPS) [10–12], the Nuclear Regulatory Authority (NRA) issued a new framework for iodine prophylaxis following nuclear accidents in 2013 [1]. This framework was revised in 2019 in accordance with revisions to the World Health Organization (WHO)’s guideline entitled “Iodine thyroid blocking (ITB)” [2]. The WHO emphasized that the group most sensitive to radioactive iodine includes children, adolescents, and pregnant and breastfeeding women. Especially, children are most likely to benefit from, and therefore should be the preferential target for, the pre-distribution of stable iodine (PDSI). Therefore, the revised points of the guideline in 2019 clearly recommended the administration of SI to individuals aged less than 40 years based on previous knowledge obtained after the Chernobyl accident [13].

In general, PDSI depends on the distance people live from a nuclear power plant. In Japan, the area within a radius of approximately 5 km of a nuclear power plant is defined as the precautionary action zone (PAZ), and precautionary urgent protective actions for preventing or mitigating the occurrence of severe deterministic effects should be prepared for these areas. In the PAZ, PDSI should be available to all residents under the age of 40. The area within a radius of approximately 5–30 km of a nuclear power plant is defined as the urgent protective action planning zone (UPZ), and protective actions for providing prompt sheltering, environmental monitoring, and implementation of urgent protective actions based on the results of environmental monitoring within a few hours after the release of radionuclides should be prepared. In the UPZ, PDSI is available only to those who have applied. For smooth implementation of ITB, SI should be stored strategically at hospitals, public health centers and local community centers in the UPZ [13, 14].

**Fig 1:**
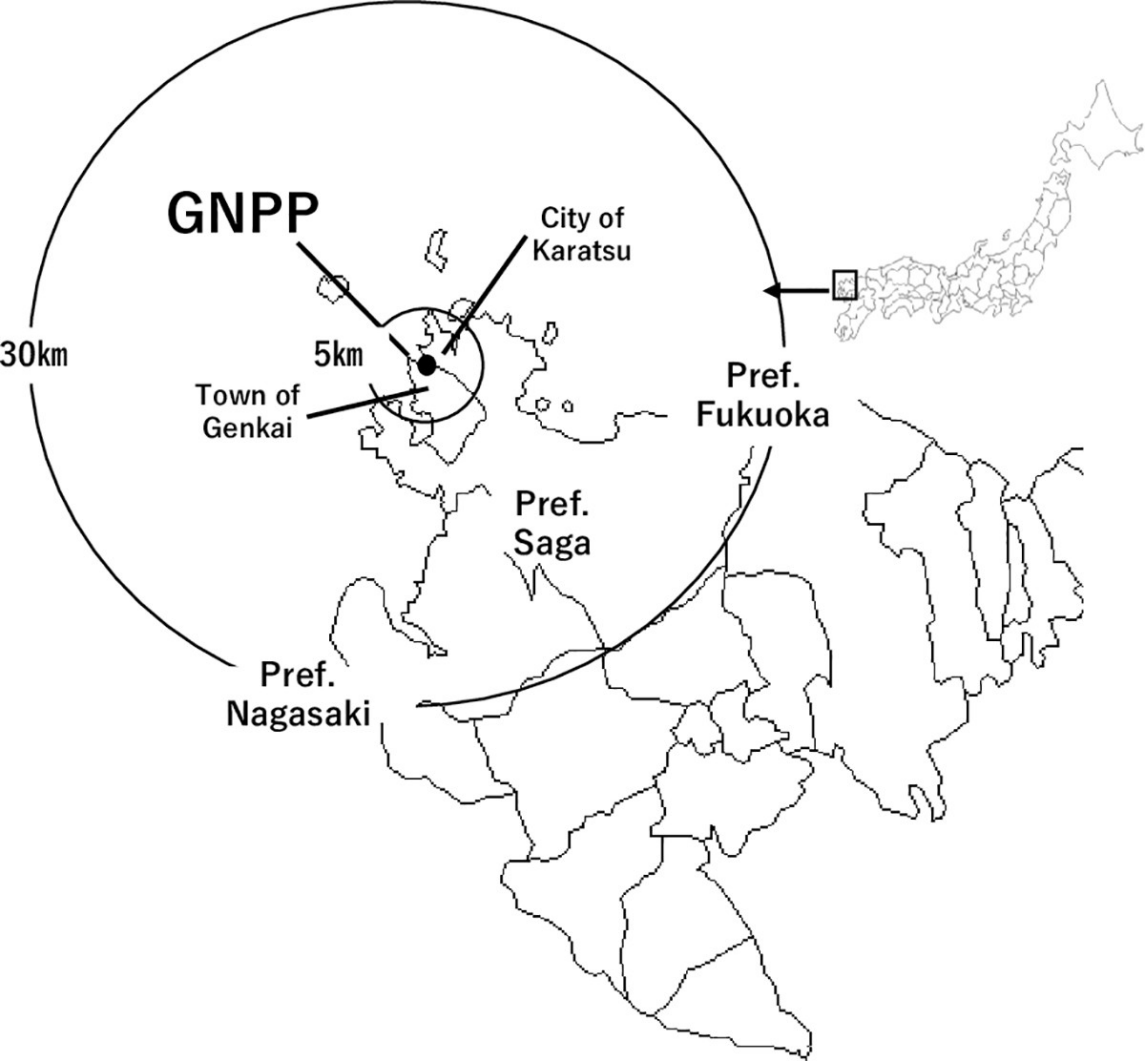
Location of the Genkai Nuclear Power Plant (GNPP) in Saga Prefecture, Japan.

Kyushu Electric Power Co., Inc.’s Genkai Nuclear Power Plant (GNPP) is located in Genkai Town, Saga Prefecture, Japan (Fig 1). After the FDNPS accident, operations at all reactors at the GNPP were stopped. In 2018, two of the four reactors were restarted in accordance with the new regulations outlined by the NRA [15]. Around the GNPP, the total population in the PAZ is 8,126 (2,876 households) people living in Genkai Town and Karatsu City, and that of the UPZ is 254,700 (103,330 households) people living in one town and seven cities in Saga, Nagasaki and Fukuoka Prefectures [16]. The updated WHO guidelines [2] stated that planning and education for PDSI to households in the vicinity of nuclear reactors should be carefully considered. In addition, each local municipality around the nuclear power plant should be responsible for the distribution of SI and instructing the public on how to use it [12]. According to these recommendations by the WHO, local municipalities in the PAZ around the GNPP organized annual meetings for residents to explain the distribution of SI and ITB [17]. In addition, Saga Prefecture published a brochure to help residents better understand procedures for evacuation, sheltering and ITB during an unexpected nuclear disaster, which was distributed to all households in the prefecture. Furthermore, prefectures that include a nuclear power plant hold annual meetings about ITB and SI [18]. Although efforts have been made to raise awareness of ITB among local residents in Saga Prefecture, their effectiveness has not been evaluated, especially among guardians of children and infants. Therefore, the objective of this study was to evaluate the risk perception of guardians with children living around the GNPP.

## Methods

### Study participants

This study was conducted from December 2019 to February 2020 in four municipalities in Saga Prefecture, Japan, including those within the PAZ and some of the UPZ of the GNPP. We distributed a self-administered questionnaire regarding perception of risks associated with administration of SI to approximately 400 guardians of children aged 0–6 in 10 kindergartens located in the four municipalities. We obtained responses from 286 guardians, and after excluding incomplete responses and those who did not live within 5 km (PAZ) or 5–30 km (UPZ) of the GNPP, 247 guardians (228 mothers, 18 fathers and one grandmother) were included in the final analysis. Prior to the study, we explained to the participants using the paper describing the study and obtained informed consent from all of them. This study was approved by the ethics committee of Nagasaki University Graduate School of Biomedical Sciences (No. 19083003).

### Data collection

The self-administered questionnaire asked guardians whether they were aware of SI. To those who answered “yes”, we asked whether they had received PDSI. We defined those who had received PDSI as the “PDSI (+)” group, and those who had not as the “PDSI (-) group”. We asked the PDSI (-) group the reason why they did not receive SI, with the following response choices: “anxiety about the side effects of SI”, “distrust of the effectiveness of SI”, “complicated procedures for receiving SI”, “missed the date for receiving SI” and “other”. Multiple answers were permitted. We also asked about demographic factors including sex, age, number of children under 18 years of age, and about social factors including distance from the GNPP to their home, i.e. within 5 km (PAZ), within 30 km (UPZ), or more than 30 km. We classified age as “under 30 y”, “30s”, “40s”, and “50 y or older” In addition, we asked guardians whether they were aware that it is preferential to implement ITB to children after a nuclear accident, whether they were aware of the booklet about prophylaxis of SI published by the local government, and whether they had used social network services (SNS) to collect information about radiation exposure. Furthermore, we asked if guardians felt anxious about administering medication to their children in general, and whether they felt anxious about administering SI to their children. For these two questions, the four response choices were “yes”, “I think so”, “I don’t think so” and “no”. We classified responses of “yes” and “I think so” as “yes”, and responses of “no” and “I don’t think so” as “no”.

### Statistical analyses

We evaluated the differences between the PDSI (+) and PDSI (-) groups using chi-square tests. Then, we identified factors independently associated with PDSI using binominal logistic regression analysis. In the binominal logistic regression analysis, we included “age”, “distance from the GNPP to the participants’ home (PAZ or UPA)”, “awareness of preferential implementation of ITB to children”, “awareness of the prophylaxis booklet published by the local government” and “anxiety about the administration of SI to children” as covariates, since the p-values obtained from the chi-square tests for these items were less than 0.05. P-values less than 0.05 were considered significant. Statistical analysis was performed using IBM SPSS Statistics Version 19 software (SPSS Japan, Tokyo).

## Results

**Fig 2:**
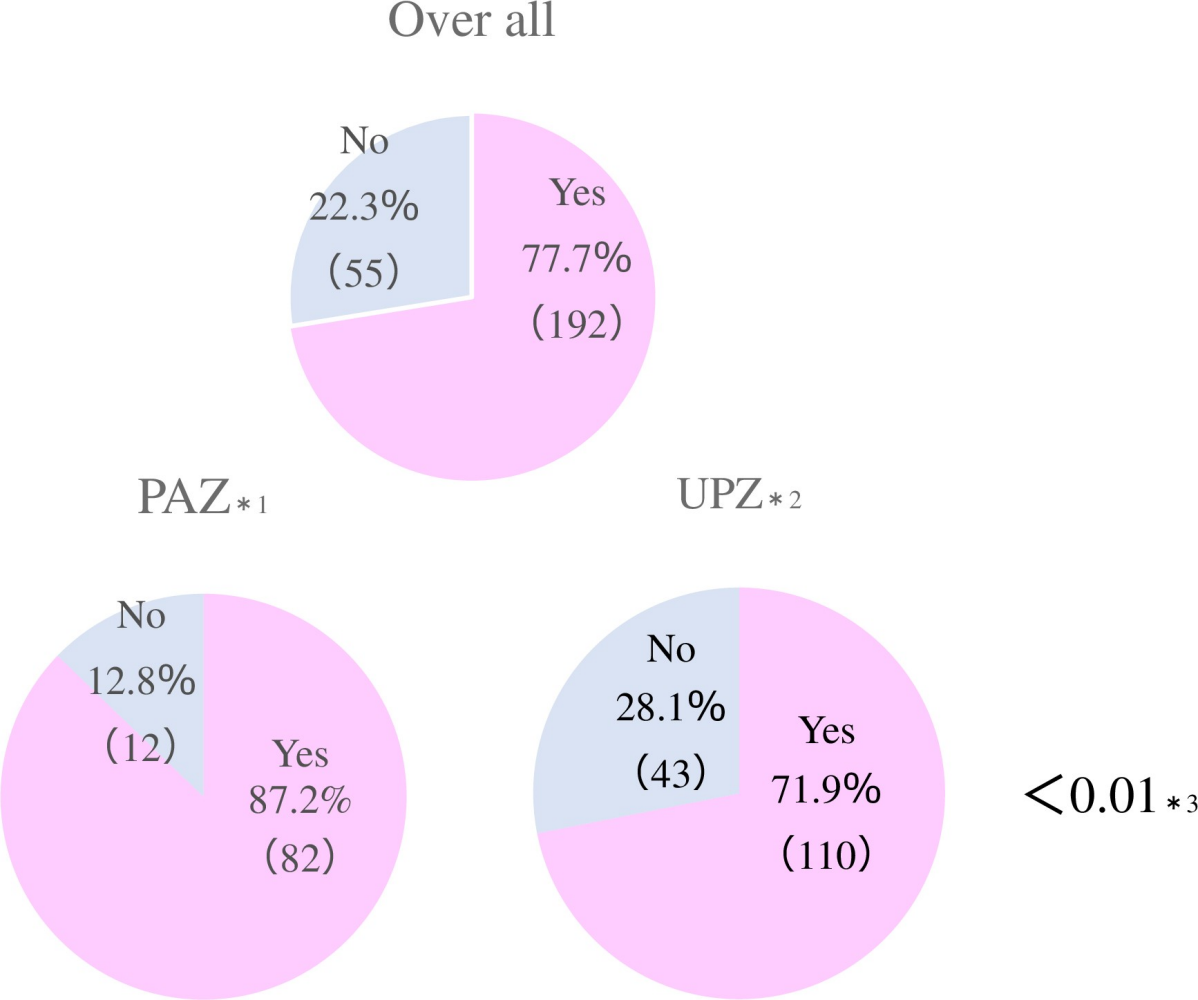
Frequency of residents in the PAZ and UPZ who were aware of SI. *1. Awareness of SI in the PAZ, *2. Awareness of SI in the UPZ, *3. Chi-square test for the PAZ and UPZ, PAZ = Precautionary Action Zone, UPZ = Urgent Protective action planning Zone.

**Fig 3:**
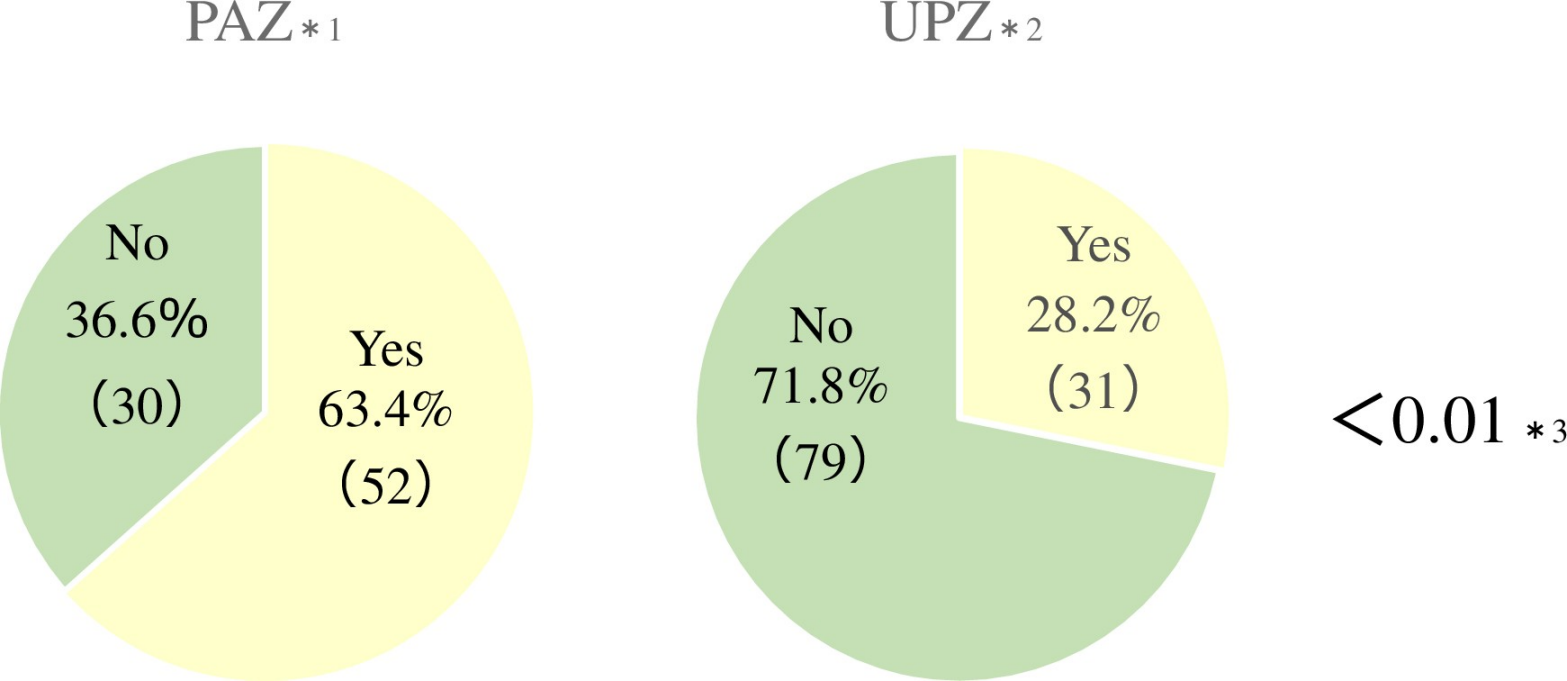
Frequencies of residents in the PAZ and UPZ who received PDSI. *1. Number who were aware of SI in the PAZ, *2. Number who were aware of SI in the UPZ, *3. Chi-square test for the PAZ and UPZ. PAZ = Precautionary Action Zone, UPZ = Urgent Protective action planning Zone, PDSI = pre-distribution of stable iodine, SI = stable iodine.

A total of 83 of 247 (33.6%) guardians comprised the PDSI (+) group, and 164 (66.4%) comprised the PDSI (-) group. 94 of 247 (38.1%) guardians lived in the PAZ, and 153 (61.9%) lived in the UPZ. 192 of 247 (77.7%) responded that they were aware of SI and 55 (22.3%) responded that they were not. 82 of 94 (87.2%) guardians living in the PAZ responded that they were aware of SI, and 110 of 153 (71.9%) guardians living in the UPZ responded that they were aware of SI (p<0.01) (Fig 2). Among the guardians who were aware of SI, 52 of 82 (63.4%) living in the PAZ and 31 of 110 (28.2%) living in the UPZ had received PDSI from the local government (p<0.01) (Fig 3).

229 of 247 (92.7%) guardians were female, 39 (15.8%) had one child, 94 (38.1%) had two children, and 114 (46.1%) had three or more children. Regarding the age of the guardians, 41 (16.6%) were under 30 y, 158 (64.0%) were in their 30s, 48 (19.0%) were in their 40s, and one (0.4%) was 50 y or older.

Table 1 shows the characteristics of the guardians in this study. Significantly more guardians in the PDSI (+) group lived in the PAZ (n = 52, 62.7%) than in the PDSI (-) group (n = 42, 25.6%) (p<0.01). Significantly more guardians in the PSDI (+) group were aware of the preferential implementation of ITB to children (n = 59, 71.7%) than in the PDSI (-) (n = 60, 36.6%) (p<0.01). Similarly, significantly more guardians were aware of the prophylaxis booklet published by the local government in the PDSI (+) group (n = 50, 60.2%) than in PDSI (-) group (n = 56, 34.1%) (p<0.01). On the other hand, the frequency of guardians with anxiety about the administration to SI to children were significantly lower in the PDSI (+) group (n = 54, 65.1%) than in the PDSI (-) group (n = 126, 76.8%) (p = 0.04). The frequency of guardians with anxiety about the administration of meditation to children in general (26.5% vs. 32.3%, p = 0.22), and those with experience using SNS to collect information about radiation exposure (20.5% vs. 22.0%, p = 0.46) were not significantly different between the PDSI (+) and PDSI (-) groups, respectively.

**Table 1:** Characteristics of the guardians.

	Total (n = 247), n (%)	PDSI (+) group (n = 83), n (%)	PDSI (-) group (n = 164), n (%)	p-value
Age under 39 years old	199 (80.6)	69 (83.1)	130 (79.3)	0.29
Living in the PAZ	94 (38.1)	52 (62.7)	42 (25.6)	<0.01
Aware of preferential implementation of ITB to children	119 (48.2)	59 (71.1)	60 (36.6)	<0.01
Aware of the prophylaxis booklet published by the local government	106 (42.9)	50 (60.2)	56 (34.1)	<0.01
Anxiety about the administration of SI to children	180 (72.9)	54 (65.1)	126 (76.8)	0.04
Anxiety about the administration of meditation to children in general	75 (30.4)	22 (26.5)	53 (32.3)	0.22
Experience using SNS to collect information about radiation exposure	53 (21.5)	17 (20.5)	36 (22.0)	0.46

Note. Chi-square tests. PDSI = pre-distribution of stable iodine, PAZ = precautionary action zone, ITB = iodine thyroid blocking, SI = stable iodine, SNS = social network services.

In the PDSI (-) group, the most common reason for not receiving PDSI was “anxiety about the side effects of SI” (40.2%), followed by “distrust of the effectiveness of SI” (23.5%), “complicated procedures for receiving SI” (15.7%) and “missed the date for receiving SI” (8.8%), respectively (Table 2).

**Table 2:** Reasons for not having received PDSI.

	n (%)
Anxiety about the side effects of SI	41 (40.2)
Distrust of the effectiveness of SI	24 (23.5)
Complicated procedures for receiving SI	16 (15.7)
Missed the date for receiving SI	9 (8.8)
Other	12 (11.8)

Note. Multiple responses were permitted. PDSI = pre-distribution of stable iodine, SI = stable iodine.

Logistic regression analysis indicated that living in the PAZ (odds ratio [OR] = 4.49, 95% confidence interval [CI]: 2.42–8.31, p<0.01), awareness of the preferential implementation of ITB to children (OR = 3.87, 95%CI: 2.01–7.44, p<0.01), and awareness of the prophylaxis booklet published by the local government (OR = 2.70, 95%CI: 1.44–5.05, P = 0.02) were independently associated with PDSI (Table 3).

**Table 3:** Logistic regression analysis of guardians who received PDSI.

Variables	Reference	OR (95% Cl)	p-/value
Age under 39 years old	Yes/No	2.19 (0.97–4.93)	0.59
Living in the PAZ	Yes/No	4.49 (2.42–8.31)	<0.01
Aware of the preferential implementation of ITB to children	Yes/No	3.87 (2.01–7.44)	<0.01
Aware of the booklet about prophylaxis of published by the local government	Yes/No	2.70 (1.44–5.05)	0.02
Anxiety about the administration of SI to children	Yes/ No	0.99 (0.50–1.99)	0.99

Note. Binominal logistic regression analysis. PDSI = pre-distribution of stable iodine, PAZ = precautionary action zone, ITB = iodine thyroid blocking, SI = stable iodine, OR = odds ratio, 95%CI = 95% confidence interval.

## Discussion

To the best of our knowledge, this is the first study to investigate the profile of guardians of children living around the PAZ of a nuclear power plant. We showed that living in the PAZ, awareness of the preferential implementation of ITB to children and awareness of a prophylaxis booklet published by the local government were independently associated with receiving PDSI.

We showed that awareness of SI was significantly higher among guardians living in the PAZ than in the UPZ. Furthermore, the frequency of guardians who had received PDSI was higher in the PAZ than in the UPZ. If a nuclear disaster were to occur at the GNPP, residents living in the PAZ who have already received SI would be able to start prophylaxis immediately after notification from the nuclear emergency headquarters or local public authorities, prior to evacuation following the permitted the evacuation route plan by the local administration. On the other hand, residents living in the UPZ would likely be instructed by the national government or local municipalities to shelter indoors, and thereafter, depending on situation of the disaster, they would receive SI from local municipalities just before evacuation, if necessary [13, 19, 20]. Such different protocols of SI prophylaxis during a nuclear disaster between residents of the PAZ and UPZ might cause differences in awareness of SI and receiving PDSI among guardians.

In this study, we showed that 36.6% of guardians living in the PAZ had not received PDSI. During a nuclear disaster, one major concern is delayed evacuation because it is time consuming to distribute SI [21]. Iodine prophylaxis blocks the uptake of ^131^I by the thyroid. Iodine prophylaxis is 98%–99% effective for blocking radioiodine if it is administered at the time of or just prior to exposure and is 85%–90% effective 1–2 h following exposure [22]. From this point of view, the low rate of PDSI is a serious problem not only in Japan, but also in other countries [23, 24].

Our study showed that awareness of the prophylaxis booklet published by the local government was associated with having received PDSI. Saga Prefecture, where the GNPP is located, published an original brochure explaining ITB and the actions that are necessary for residents to take during a nuclear disaster. Furthermore, the booklet contained information and explanations about the evacuation routes in the PAZ and UPZ areas to shelters outside of a 30-km radius from the GNPP [18]. According to the new guidelines for iodine prophylaxis issued by the NRA, PDSI will be available to residents living in the PAZ [24]. In addition, in some areas around nuclear power plants in Japan including the GNPP, PDSI is also available to residents living in the UPZ who request it [13, 25]. In view of this situation, it is important to have risk communication between local authorities, specialists, and residents with respect to ITB and PDSI, not only in the PAZ but also in the UPZ.

We also showed that awareness of preferential implementation of ITB to children was independently associated with PDSI. According to the lessons learned from the accident at the Chernobyl Nuclear Power Plant, children are the most vulnerable to internal radiation exposure by radioiodine during a nuclear disaster [26–28]. For the smooth implementation of ITB during an unexpected nuclear disaster, PDSI, especially for children, is very important. On the other hand, we showed that the main reason why PDSI (-) guardians had not received PDSI was “anxiety about the side effects of SI”. After the accident at the FDNPS, due to the prompt evacuation and food regulation policy implemented by Japanese Government, thyroid doses are estimated to be relatively limited [29]. Actually, thyroid monitoring just after the accident in Fukushima Prefecture revealed that no children showed a level greater than 100 mSv and the highest level was less than 50 mSv [30]. These results suggest that iodine prophylaxis was not absolutely necessary during the accident. Nevertheless, anxieties about iodine prophylaxis including its side effects were observed especially in parents. Iodine prophylaxis is usually a single administration, and its side effects, such as skin rash and gastrointestinal discomfort are relatively rare, according to experiences in Poland which implemented iodine prophylaxis after the Chernobyl accident [31, 32]. Prior to PDSI, it is important for local authorities and specialists to communicate with guardians to explain the pros and cons of SI.

There are several limitations in this study. First, we conducted this study only in the PAZ/UPZ around the GNPP. We need to expand the study area to inside and outside Japan. Also, this study was conducted just after the commencement of PDSI around the UPZ in Japan. Longitudinal studies are needed, according to changes in the situation of PDSI in Japan.

In conclusion, we showed that living in the PAZ, awareness of the preferential implementation of ITB to children and awareness of the prophylaxis booklet published by the local government were independently associated with having received PDSI in guardians of children living around the PAZ of a nuclear power plant. For the effective implementation of ITB during an unexpected nuclear disaster, it is necessary to clarify the risk perception in guardians, and to continue risk communication.

## Supporting information

S1 Data (XLSX)

S1 Questionnaire (DOCX)

## References

[1] World Health Organization, Guidelines for iodine prophylaxis following nuclear accidents 1999. Available at https://www.who.int/ionizing_radiation/pub_meet/Iodine_Prophylaxis_guide.pdf. Accessed 2020 June 26.

[2] World Health Organization, Iodine thyroid blocking guidelines for use in planning and responding to radiological and nuclear emergencies. Available at https://www.who.int/ionizing_radiation/pub_meet/iodine-thyroid-blocking/en/. Accessed 2020 October 10. 29630192

[3] Jang M, Kim HK, Choi CW, Kang CS. Age-dependent potassium iodide effect on the thyroid irradiation by 131I and 133I in the nuclear emergency. Radiat Prot Dosimetry. 2008;130(4):499–502. 10.1093/rpd/ncn068 18337292

[4] Rooney AA, Cooper GS, Jahnke GD, Lam J, Morgan RL, Boyles AL, et al. How credible are the study results? Evaluating and applying internal validity tools to literature-based assessments of environmental health hazards. Environment Internat. 2016;92–93:617–629. 10.1016/j.envint.2016.01.005 26857180 PMC4902751

[5] Thompson DE, Mabuchi K, Ron E, Soda M, Tokunaga M, Ochikubo S, et al. Cancer incidence in atomic bomb survivors. Part II: Solid tumors, 1958–1987. Radiation Research. 1994;137: S17–S67. 8127952

[6] Schneider AB, Sarne DH. Long-term risks for thyroid cancer and other neoplasms after exposure to radiation. Nat Clin Pract Endocrinol Metab. 2005;1(2):82–91. 10.1038/ncpendmet0022 16929376

[7] The Chernobyl Forum: 2003–2005. Chernobyl’s legacy: Health, environmental and socio-economic impacts. Vienna: International Atomic Energy Agency, 2006.

[8] International Atomic Energy Agency. IAEA Safety standards for protecting people and the environment. Preparedness and response for a nuclear or radiological emergency: general safety requirements part 7. Available at https://www-pub.iaea.org/MTCD/publications/PDF/P_1708_web.pdf. Accessed 2020 June 26.

[9] International Atomic Energy Agency. IAEA Safety standards for protecting people and the environment. Criteria for use in preparedness and response for a nuclear or radiological emergency: general safety guide No. GSG-2. Available at https://www-pub.iaea.org/MTCD/publications/PDF/Pub1467_web.pdf. Accessed 2020 June 26.

[10] Nuclear Emergency Response Headquarters of Japanese Government. Report of Japanese Government to the IAEA Ministerial Conference on Nuclear Safety: The accident at TEPCO’s Fukushima Nuclear Power Stations. Available at http://japan.kantei.go.jp/kan/topics/201106/iaea_houkokusho_e.html. Accessed 2020 June 20.

[11] Nuclear Emergency Response Headquarters of Japanese Government. Additional report of the Japanese Government to the IAEA—The accident at TEPCO’s Fukushima Nuclear Power Stations, 15 September 2011. Available at https://www.iaea.org/newscenter/focus/fukushima/additional-japan-report. Accessed 2020 June 20.

[12] International Atomic Energy Agency. The Fukushima Daiichi Accident, Report by the Director General. Available at https://www-pub.iaea.org/MTCD/Publications/PDF/Pub1710-ReportByTheDG-Web.pdf. Accessed 2020 June 20.

[13] Nuclear Regulation Agency. For distribution and prophylaxis of stable iodine. Available at https://www.nsr.go.jp/data/000024657.pdf. Accessed 2020. Accessed 2020 June 20.

[14] Nuclear Regulation Authority. Nuclear Emergency Response Guidelines. Available at https://www.nsr.go.jp/data/000300735.pdf. Accessed 2020 June 20. (In Japanese).

[15] Nuclear Regulation Authority. Genkai NRA Regional Office. Available at https://www.nsr.go.jp/english/nuclearfacilities/genkai/index.html. Accessed 2020 June 20.

[16] Cabinet Office, Government of Japan. Emergency response in the Genkai area. Available at https://www8.cao.go.jp/genshiryoku_bousai/keikaku/02_genkai.html. Accessed 2020 June 26. (in Japanese).

[17] Saga Prefectural Government Office. We carry out Saga atomic energy disaster prevention drills in 2019. Available at https://www.pref.saga.lg.jp.e.zg.hp.transer.com/bousai/kiji00371787/index.html. Accessed 2020 June 26.

[18] Saga Prefectural Government Office. Creation of the Nuclear Disaster Prevention Handbook, Available at https://www.pref.saga.lg.jp/bousai/kiji00364494/index.html#. Accessed 2020 June 26.

[19] Saga Prefectural Government Office. Saga Prefecture regional disaster prevention planning. Available at https://www.pref.saga.lg.jp/kiji00361211/3_61211_135741_up_tmwl6dex.pdf. Accessed 2020 June 26. (In Japanese).

[20] Yoshida S, Ojino M, Ozaki T, Hatanaka T, Nomura K, Ishi M, et al. Guidelines for iodine prophylaxis as a protective measure: Information for physicians. JMJA. 2014;57(3):113–123. 25784824 PMC4356652

[21] Adalja AA, Sell TK, Ravi SJ, Minton K, Morhard R. Emergency Preparedness in the 10-Mile Emergency Planning Zone Surrounding Nuclear Power Plants. J Homel Secur Emerg Manag. 2014;12(1):81–100. 10.1515/jhsem-2013-0106 26692825 PMC4676567

[22] Braverman ER, Blum K, Loeffke B, Baker R, Kreuk F, Yang SP, et al. Managing Terrorism or Accidental Nuclear Errors, Preparing for iodine-131 Emergencies: A Comprehensive Review. Int J Environ Res Public Health. 2014;11(8):7803–7804. 10.3390/ijerph110404158 24739768 PMC4025043

[23] Zwolinski LR, Stanbury M, Manente S. Nuclear Power Plant Emergency Preparedness: Results from an Evaluation of Michigan’s Potassium Iodide Distribution Program. Disaster Med Public Health Prep. 2012;6(3):263–269. 10.1001/dmp.2012.41 23077269

[24] Ojino M, Yoshida S, Nagata T, Ishii M. First Successful Pre-Distribution of Stable Iodine Tablets Under Japan’s New Policy After the Fukushima Daiichi Nuclear Accident. Disaster Med Public Health Prep. 2017;11(3):365–369. 10.1017/dmp.2016.125 27927262

[25] Cabinet Office, Government of Japan. Points for “Operations related to PDSI agents”. Available at https://www8.cao.go.jp/genshiryoku_bousai/pdf/08_sonota_yosozai.pdf. Accessed 2020 June 26. (In Japanese).

[26] Kazakov VS, Demidchik EP, Larisa N. Astakhova. Thyroid cancer after Chernobyl. Nature. 1992;359:21–22. 10.1038/359021a0 1522879

[27] Heidenreich WF, Kenigsberg J, Jacob P, Buglova E, Goulko G, Paretzke HG, et al. Time trends of thyroid cancer incidence in Belarus after the Chernobyl Accident. Radiation Research. 1999;151:617–625. 10319735

[28] Astakhova LN, Anspaugh LR, Beebe GW, Bouville A, Drozdovitch VV, Garber V, et al. Chernobyl-related thyroid cancer in children of Belarus: a case-control study. Radiation Research. 1998;150:349–356. 9728663

[29] United Nations Scientific Committee on the Effects of Atomic Radiation (UNSCEAR). UNSCEAR 2013 report volume I. Report to the general assembly scientific annex A: Levels and effects of radiation exposure due to the nuclear accident after the 2011 great east-Japan earthquake and tsunami. Available at: http://www.unscear.org/docs/reports/2013/13-85418_Report_2013_Annex_A.pdf. Accessed 3 December 2015. Accessed 2020 October 10.

[30] Nagataki S, Takamura N. Radioactive Doses—Predicted and Actual—and Likely Health Effects. Clin Oncol (R Coll Radiol). 201;28:245–254. 10.1016/j.clon.2015.12.028 26805911

[31] Becker DV, Zanzonico P. Potassium iodide for thyroid blockade in a reactor accident: Administrative policies that govern its use. Thyroid. 1997;7:193–197. 10.1089/thy.1997.7.193 9133683

[32] Nauman J., Wolff J. Iodide prophylaxis in Poland after the Chernobyl reactor accident: Benefits and risks. Am J Med. 1993;94:524–532. 10.1016/0002-9343(93)90089-8 8498398

