# Supplementary material for: Risk perception of the pre-distribution of stable iodine to guardians of children living around the Genkai Nuclear Power Plant, Saga Prefecture, Japan
Source: PLoS One. 2021 May 13;16(5):e0250570. doi: 10.1371/journal.pone.0250570 (PMC8118537; doi:10.1371/journal.pone.0250570)
Supplement: S1 Questionnaire — (DOCX) [file pone.0250570.s002.docx]

**S1 Questionnaire.**

Please answer the following questions.

1. What is your gender?

i) male ii) female

1. How old are you?

i) under 30 y ii) 30s iii) 40s iv) 50 y or older

1. How many children under 18 years old you are living with?

supplementally i) one　　ii) two iii) more than three

1. What is relationship of you with your child/children?

i) mother ii) father　　iii) grandparents iv) others

1. What is your current living area?

i) within 5km from GNPP (PAZ) ii) within 5-30km from GNPP (UPZ)

iii) over 30km from GNPP iv) unknown

1. Have you ever felt anxieties when you administrate general medicine to children?

i) yes ii) probably yes　　iii) probably no iv) no

1. Do you know the booklet about prophylaxis of SI published by the local government?

i) yes ii) no

1. Have you ever used social network services (SNS) to collect information about radiation exposure?

i) yes ii) no

1. Do you know the SI before answering this questionnaire?

i) yes ii) no

↓

Please answer those who answered “yes”.

1. Do you know the preferential implementation of ITB to children after a nuclear accident?

i) yes ii) no

1. Have you ever received pre-distributed SI?

i) yes ii) no

1. Do you want to receive pre-distributed SI?

i) yes ii) no

↓

Please answer only those who answered “no”

1. Why do you not want to receive predistribution of SI?
   1. anxieties about the side effects of SI
   2. distrust to the effectiveness of SI
   3. complicated procedures for receiving SI
   4. missed the date for receiving SI
   5. other
2. Do you feel anxieties for the administration of SI to your children?

i) yes ii) no

Thank you.
